# Hydraulisch geregelte isokinetische Krafttestung

**DOI:** 10.1007/s00132-025-04670-3

**Published:** 2025-07-17

**Authors:** Jan Schröder, Miriam Knauer, Gunnar Liedtke

**Affiliations:** Institut für Bewegungswissenschaft, Universität Hamburg, Turmweg 2, Hamburg, Deutschland

**Keywords:** Referenzwerte, Drehmoment, Isometrische Kontraktion, Muskelkraft, Querschnittsanalyse, Reference values, Torque, Isometric contraction, Muscle strength, Cross sectional analysis

## Abstract

**Hintergrund:**

Die hydraulisch geregelte Widerstandsgebung kann als Sonderform der Isokinetik betrachtet werden, weil eine konstante Bewegungsgeschwindigkeit vorgegeben werden kann. Für diese spezielle Krafttestvariante liegt wenig vergleichende Literatur vor. In dieser Arbeit werden für die hydraulisch geregelte (Quasi‑)Isokinetik Orientierungswerte für Rumpf- und Kniekräfte und deren funktionelle Quotienten vorgestellt und die Reproduzierbarkeit und Vergleichbarkeit mit der Isometrie evaluiert.

**Methodik:**

In einer Querschnittstudie wurden für 45 gesunde Erwachsene (21 Frauen, Alter 26,1 ± 3,9 Jahre, BMI 23,2 ± 2,5 kg/m^2^) Orientierungswerte (M, SD, Perzentile) für die isometrische und isokinetische Maximalkraft und funktionelle Quotienten (Flexion, Extension: Rumpf, Knie) für die Factum® Diagnosesysteme (Frei medical) ermittelt und miteinander vergleichen (Bland-Altman). Zeitaufwand und Reliabilität wurden für eine Teilstichprobe (50 % Frauen) überprüft (ICC3.k).

**Ergebnisse:**

Die (Quasi‑)Isokinetik war zeitökonomischer (ca. 50 % Zeitaufwand) und tendenziell reliabler (ICC3.k 0,736–0,933) als die Isometrie (ICC3.k 0,550–0,899). Funktionelle Flexions-Extensions-Quotienten (Isokinetik 68 %, Isometrie Rumpf 63 %) wiesen in etwa eine 2:3-Relation auf, außer beim Isometrie-Knie-Quotienten (55 %, ca. 1:2), wo die Flexion in Relation schwächer in den Quotienten einging.

**Diskussion:**

Die Testergebnisse für die isometrischen und (quasi‑)isokinetischen Krafttests sind nicht miteinander vergleichbar, was insbesondere bei den funktionellen Quotienten für die Praxis beachtet werden muss. Jedes System für sich ist reliabel, die Isokinetik hatte eine bessere Testökonomie. Die gerätespezifischen auf das Körpergewicht relativierten Orientierungswerte sind für die Praxis nützlich.

**Graphic abstract:**

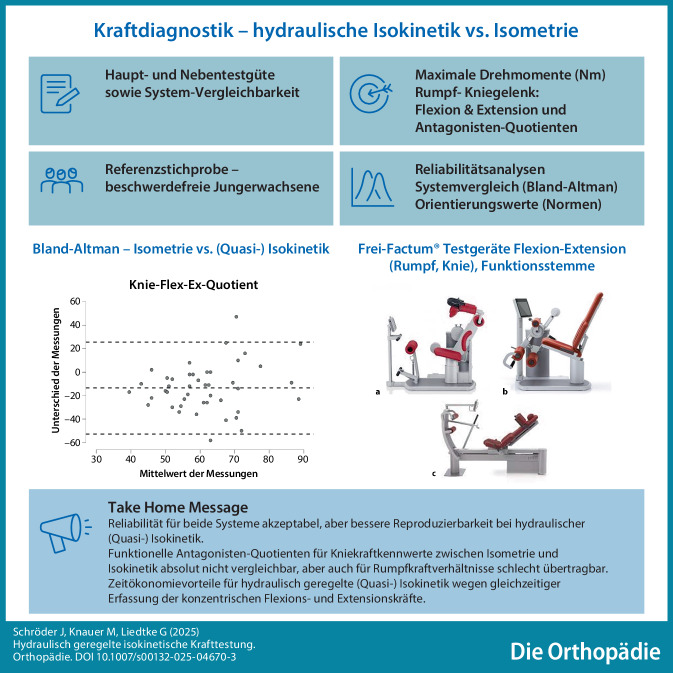

**Zusatzmaterial online:**

Die Online-Version dieses Beitrags (10.1007/s00132-025-04670-3) enthält umfangreiches Zusatzmaterial.

## Abstract (en)

**Background:**

Hydraulically controlled strength test devices provide a constant predetermined movement velocity, and as such may be specified as (quasi‑)isokinetic dynamometry. There are limited literature reporting comparisons with other strength testing modes. This work presents reference values and reliability analyses for trunk and knee maximum torques and their respective flexion-extension ratios for hydraulically controlled isokinetic dynamometry in comparison to isometric strength testing.

**Materials and methods:**

In a cross-sectional design, reference values (M, SD, percentiles) of 45 healthy adults (21 females, age 26.1 ± 3.9 years, BMI 23.2 ± 2.5 kg/m^2^) were assessed (trunk, knee: flexion, extension and their functional ratios) for the Factum® test devices (Frei medical) including, comparisons of the isometric or isokinetic test mode (Bland-Altman). For a sub-sample of 20 persons (50% females), time economy and reliability (ICC3.k) were assessed.

**Results:**

The (quasi‑)isokinetic test protocol was less time consuming (50%) and demonstrated higher reliability coefficients (ICC3.k 0.736–0.933) compared to isometric testing (ICC3.k 0.550–0.899). Flexion-extension ratios (isokinetic 68%, isometric trunk 63%) revealed a relation nearby 2:3, except for the isometric knee ratio (55% ≈ 1:2), due to smaller proportions of knee flexion forces.

**Conclusion:**

Results indicated mode specific differences and are not directly comparable with clinical implications especially for the flexion-extension ratios. Each protocol showed to be sufficiently reliable with time economy advantages for the (quasi‑)isokinetic protocol. Distributions of body weight-adjusted peak torques and functional ratios may serve as device-specific benchmark values for strength testing in clinical environments.

Isokinetik gilt weiterhin als Goldstandard in der Kraftdiagnostik zur Qualitätssicherung im Monitoring orthopädischer Rehabilitation, obwohl für die Praxis bedeutsame testökonomische Aspekte eher für isometrische Testgeräte sprechen. Diese Nachteile beim Personal‑, Finanz- und Zeitaufwand gelten nicht im gleichen Maße für eine hydraulisch geregelte (Quasi‑)Isokinetik, für die jedoch bislang wenig empirische Befunde zur Testgüte vorliegen. Orientierungswerte, Reproduzierbarkeit und die Vergleichbarkeit mit der Isometrie, sowie testökonomische Aspekte werden in dieser Arbeit vorgestellt.

Die Grundlagen für die heutige Trainingstherapie in der orthopädischen Rehabilitation und der apparativen Qualitätssicherung basieren auf empirischen Befunden aus den 1950er-Jahren, die einerseits Muskelkraft- und Funktionsdefizite bei Rückenbeschwerden beschreiben, andererseits aber auch positive Effekte von Trainingsmaßnahmen aufzeigen [1–3], indem seit den 1980er-Jahren Rumpfmuskelkraftprofile in allen Dimensionen bei Rückenschmerzpatienten im Vergleich mit Gesunden erhoben wurden [4, 5]. Neben der isokinetischen Kraftdiagnostik ist die apparativ einfachere Isometrie Bestandteil der Qualitätssicherung in der Rehabilitation muskuloskelettaler Erkrankungen, obwohl bei der Isometrie – im Gegensatz zur Isokinetik – die direkte Ableitung von Trainingslasten und -intensitäten aus den objektivierenden Krafttestungen entkoppelt ist [6–9]. Für jede Form der Krafttestung muss sichergestellt sein, dass die erhobenen Kraftkennwerte nicht nur wissenschaftlichen Hauptgütekriterien (Validität, Reliabilität, Objektivität) genügen, sondern auch Nebengütekriterien (Nützlichkeit), sodass Messwerte vor dem Hintergrund gerätespezifischer Referenz- oder Orientierungswerte interpretiert werden können [10, 11]. Die Anordnung biomechanischer Standardisierungsmaßnahmen (Polsterungen, Haltegriffe, Hebellängen oder Winkelstellungen) hat erhebliche Auswirkungen auf die realisierbaren Maximalkraftkennwerte, sodass für jeden Gerätetyp – trotz oberflächlich betrachtet bestehender Ähnlichkeiten – eigene Orientierungswerte notwendig sind [7, 12, 13]. Für die Krafttrainings- und Diagnosegeräte der Firma Frei medical (Kirchzarten, Deutschland) liegen zwar erste Orientierungswerte und Reliabilitätsanalysen für die isometrische Testung der Rumpf- sowie für die einbeinige Knieflexion und -extension vor [7], jedoch nicht für die quasi-isokinetische Testung der hydraulisch gebremsten, konzentrischen Mehrwiederholungsmaximalkrafttestung.

Eine aktuelle Übersichtsarbeit ergab, dass sich Isometrie und elektromagnetisch geregelte (klassische) Isokinetik in der Testreproduzierbarkeit nicht wesentlich unterscheiden, dass es aber im Hinblick auf die Testökonomie Unterschiede mit Vorteilen für die Isometrie gibt [6], sodass sich auch hieraus eine Forschungslücke für die spezielle, hydraulisch gebremste Isokinetik ergibt.

Ziel der vorliegenden Arbeit war es, nach Sicherung der jeweiligen Reproduzierbarkeit die Vergleichbarkeit der isometrischen und quasi-isokinetischen Krafttestungen zu evaluieren und erste Orientierungswerte für diese spezifischen Geräte vorzustellen, wobei auch das Nebengütekriterium der Zeitökonomie berücksichtigt werden soll, um diese Erkenntnisse der klinischen Nutzung und Versorgungsforschung zur Verfügung zu stellen.

## Methodik

### Design

Im Sinne einer Querschnittstudie wurden Probanden für Systemvergleiche und Orientierungswerte an einem Tag getestet. Mit einer Teilstichprobe wurde eine Test-Retest-Reliabilitätsstudie durchgeführt (7 Tage Abstand, annähernd gleiche Tageszeit).

### Stichprobe

Probanden wurden im Umfeld unseres Instituts rekrutiert, über Studienziele und -methoden informiert, haben ihre freiwillige Teilnahme attestiert und mussten lediglich orthopädisch beschwerdefrei sein. Weitere Ausschlusskriterien wurden für die vorliegende junge, sportliche Klientel nicht formuliert. Von zunächst 50 akquirierten Freiwilligen mussten die Datensätze von 5 Personen nachträglich exkludiert werden, da entgegen der Vorauskünfte doch relevante Einschränkungen eingeräumt wurden.

Die Stichprobenkennwerte der verbliebenen 45 Probanden (21 Frauen, 24 Männer, Alter 18–35 Jahre, BMI 19,3–29,9 kg/m^2^) für den Vergleich der Testsysteme und die Orientierungswerte sind tabellarisch aufbereitet (Tab. 1, oben). Die Stichprobenkennwerte für die Reliabilitätsanalyse (*n* = 20, 50 % Frauen) liegen separat vor (Tab. 1, unten).

**Tab. 1:** Stichprobenkennwerte für die Voll- (oben) und Teilstichprobe (unten)

		Alter (J)	KM (kg)	KH (m)	BMI (kg/m^2^)
*Orientierungswerte*	Frauen (*n* = 21)	25,1	62,0	1,68	21,7
	(SD)	3,6	6,8	0,61	1,4
	Männer (*n* = 24)	27,1	80,9	1,82	24,4
	(SD)	3,9	10,8	0,83	2,5
	*p* (t-Test)	0,079	< 0,001	< 0,001	< 0,001
	Gesamt (*n* = 45)	26,1	72,1	1,75	23,2
	(SD)	3,9	13,1	0,97	2,5
*Reliabilitätsprüfung*	Frauen (*n* = 10)	24,6	61,5	1,67	22,0
	(SD)	2,7	5,9	0,05	1,6
	Männer (*n* = 10)	26,2	84,9	1,86	24,6
	(SD)	2,9	6,4	0,07	2,0
	*p* (t-Test)	0,219	< 0,001	< 0,001	0,005
	Teilstichprobe (*n* = 20)	25,4	73,2	1,77	23,3
	(SD)	2,9	13,4	0,11	2,2

*KM* Körpermasse, *KH* Körperhöhe, *BMI* Body-Mass-Index

### Testinstrumente

**Abb. 1:**
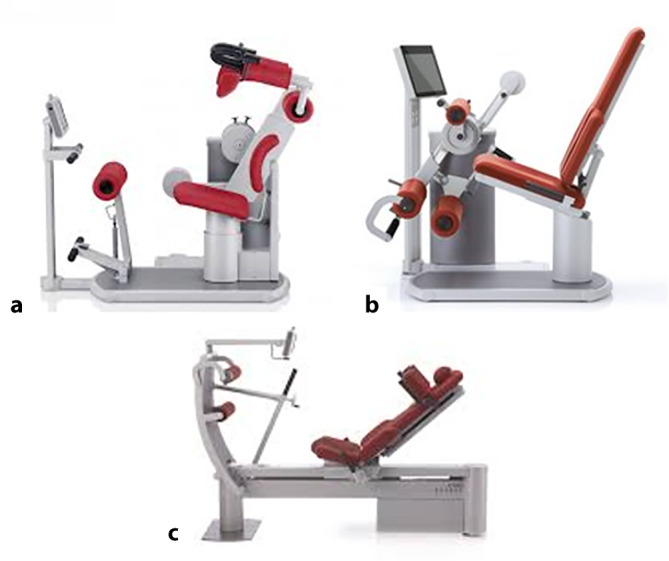
Frei-Factum®-Geräte (Kirchzarten, Deutschland) für die Testung der Rumpf-Flexion-Extension (**a**), der isolierten Knie-Flexion-Extension (**b**) und der komplexen Hüft-Knie-Streckung in der Funktionsstemme (**c**) (https://www.frei-ag.de; mit freundlicher Genehmigung des Herstellers Frei medical)

Für die Testung der Rumpf- und Knieflexion und -extension wurden Geräte der Serie Factum® (Frei medical, Kirchzarten, Deutschland) genutzt, mit denen sowohl isometrische als auch hydraulisch-geregelte, isokinetische Krafttests durchgeführt werden können. Die Testung der Knie- und Hüftstrecker (Funktionsstemme) war nur isometrisch möglich (Abb. 1).

#### Isometrie

Die isometrische Muskelspannung wird mithilfe von firmenseitig kalibrierten Druckdosen und Scherkraftwegezellen auf Basis von DMS-Messstreifen (350 Ω) mit einer Abtastrate von 100 Hz erfasst (Fehlertoleranz ± 5 % bei Null-Offset vor jeder Messung). Die Testung der Rumpfstreckung und Rumpfbeugung erfolgte mit progredienter Muskelanspannung (Plateau 2–3 sec) aus einem Startwinkel von 10° Oberkörpervorneigung. Durch Einrichtung der Sitzhöhe lag die Gerätedrehachse auf Höhe des Beckenkamms und die Unterschenkel wurden so fixiert, dass die Oberschenkel parallel zur Sitzfläche positioniert waren (Details hierzu: *Supplement 1*). Für die beidbeinige Kniestreckung wurde ein Testwinkel von 80° (spitzer Kniewinkel) und für die Kniebeugung ein Testwinkel von 50° (stumpfer Kniewinkel) – jeweils in Relation zum rechtwinklig gebeugten Kniegelenk – gewählt (Details hierzu: *Supplement 1*). In einer Funktionsstemme wurde die isometrische Maximalkraft unilateral separat für die linke und rechte Extremität mit einem eingeschlossenen Kniewinkel von 100° ermittelt (Details hierzu: *Supplement 1*). Alle isometrischen Tests wurden als Doppelmessungen – mit vorgeschalteten kurzen Probekontraktionen – durchgeführt, wobei der bessere Versuch gewertet (isometrische Maximalkraft, Nm) und der statistischen Analyse zugeführt wurde.

#### Isokinetik

Da Factum®-Geräte sowohl für isometrische als auch für isokinetische Testungen ausgelegt sind, gleichen sich die biomechanischen Rahmenbedingungen und die messtechnischen Merkmale (DMS, 350 Ω, 100 Hz Abtastrate). Die isokinetischen Testbewegungen wurden ausschließlich konzentrisch-überwindend gegen einen hydraulischen Widerstand ausgeübt, der eine voreingestellte Bewegungsgeschwindigkeit von etwa 60°/sec zuließ. Nach 4 Probekontraktionen wurden erneut 4 maximalkräftige Bewegungszyklen durchgeführt (konzentrische Beugung und Streckung), wobei das arithmetische Mittel aus diesen Zyklen (dynamisch-isokinetische Maximalkraft, Nm) der statistischen Analyse zugeführt wurde^1^.

Die Positionierung für die konzentrisch-isokinetischen Testbewegungen entsprachen den Bedingungen der Isometrie (Rumpf: Drehachse in Höhe des Beckenkamms, Oberschenkel parallel zur Sitzfläche; Knie: Drehachsenübereinstimmung zwischen Gelenk und Gerät) (siehe: *Supplement 1*).

### Testprotokoll

**Abb. 2:**
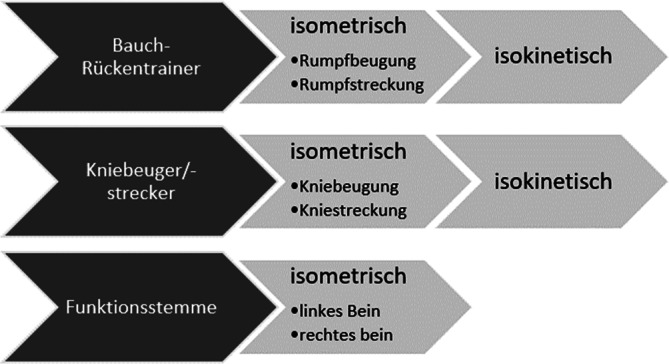
Testprotokoll

Mit Hinblick auf die notwendige Bedingungskonstanz und Standardisierung für die Reliabilitätsprüfung wurden die Probanden nach einem 5‑minütigen Fahrradergometeraufwärmen in festgelegter Reihenfolge (Isometrie gefolgt von Isokinetik: Rumpfflexion/-extension, isolierte Knieflexion/-extension, ausschließlich isometrisch komplexe Hüft-Knie-Streckung) von immer den gleichen Testleitern durch die Testsequenz begleitet (Abb. 2). Die Pausengestaltung zwischen Probekontraktionen und realem Test (ca. 10 sec: Isometrie und Isokinetik), sowie zwischen den Testwiederholungen (Isometrie: 1 min) wurden so dimensioniert, dass sich keine kumulierten Ermüdungseffekte mit Auswirkungen auf die jeweils nachfolgenden Tests ergaben [7].

Die gesamte Testbatterie dauerte 20 min, sodass die Probanden inkl. Rüstzeiten für insgesamt max. 30 min in Anspruch genommen wurden. Die Dauer der Krafttestungen wurde vom Untersucherteam erfasst (einfache Stoppuhr).

### Statistische Methoden

Die Analysen wurden mithilfe der R‑basierten Statistik-Freeware JASP (Universität Amsterdam, Niederlande) durchgeführt. Die Daten wurden parametrisch durch Mittelwert (*M*) und Standardabweichung (*SD*) beschrieben. Für Orientierungswerte wurden auch nonparametrische Kennwerte berechnet (Median, Quartile, 5 und 95 % Randperzentile). Gruppenunterschiede wurden nach Prüfung auf Normalverteilung (Shapiro-Wilk-Test) mithilfe des Student’s *t*-Test geprüft. Zur Prüfung der „*relativen*“ Reliabilität wurde der ICC_3.k_ (Intra-Klassen-Korrelationskoeffizient, ± 95 % CI) berechnet. Für die „*absolute*“ Reliabilität wurde der individuell aus Test und Retest berechnete Variationskoeffizient ($VK{\%}=\frac{SD}{M\times 100})$ sowie der Standardmessfehler $(SEM=\frac{SD}{\sqrt \mathrm{n}}$) bestimmt. Für Systemvergleiche wurden Bland-Altman-Analysen (Bias ± LoA, Limits-of-Agreement) gerechnet und graphisch illustriert. Als Signifikanzniveau wurde *p* ≤ 0,05 festgelegt.

## Ergebnisse

Für die Testung der Reliabilität der Rumpf- und Kniekraftdiagnostik ergaben sich isokinetisch höhere Intraklassenkorrelationskoeffizienten für Beugung und Streckung (ICC_3.k_ 0,736–0,933) als isometrisch (ICC_3.k_ 0,606–0,899). Die einbeinige Funktionsstemme ergab die höchsten Koeffizienten (ICC_3.k_ > 0,900). Die Reliabilität der Antagonisten-Quotienten (Flex/Ex) war für die Rumpftestungen hoch (ICC_3.k_ > 0,850), für die isometrisch ermittelten Knieantagonisten und Symmetriequotienten niedriger (ICC_3.k_ 0,550–0,651) (Tab. 2).

**Tab. 2:** Reliabilität für Isometrie (oben) und Isokinetik (unten)

		t1		t2		Test-Retest-Reliabilität				
		*M*	*SD*	*M*	*SD*	VK%	SEM	ICC 3.k	Lower	Upper
Isometrie	Rumpfflexion (Nm)	214,3	55,3	219,3	66,0	7,5	7,1	0,899	0,744	0,960
	Rumpfextension (Nm)	342,9	101,5	380,2	99,6	9,2	16,3	0,791	0,473	0,917
	Rumpf-Flex/Ex-Quotient (%)	67,2	22,8	59,4	18,1	9,3	4,0	0,888	0,716	0,955
	Knieflexion (Nm)	188,1	54,6	207,2	61,4	9,8	9,3	0,867	0,663	0,947
	Knieextension (Nm)	407,1	141,0	400,6	114,4	11,8	23,6	0,606	0,004	0,844
	Knie-Flex/Ex-Quotient (%)	48,9	15,8	53,8	19,1	12,4	4,9	0,550	−0,138	0,822
	Beinstoß (li) (Nm)	1600,3	499,1	1622,9	419,0	6,2	45,8	0,952	0,880	0,981
	Beinstoß (re) (Nm)	1676,7	492,6	1749,1	449,0	7,0	56,6	0,921	0,801	0,969
	Symmetrie-Quotient (%)	11,2	9,5	7,1	5,9	40,8	2,7	0,651	0,119	0,862
Isokinetik	Rumpfflexion (Nm)	179,9	51,2	186,1	56,5	6,8	8,2	0,912	0,778	0,965
	Rumpfextension (Nm)	286,1	79,0	288,8	65,8	7,5	14,7	0,736	0,333	0,896
	Rumpf-Flex/Ex-Quotient (%)	65,1	20,1	65,0	18,9	7,4	3,5	0,878	0,692	0,952
	Knieflexion (Nm)	168,0	45,8	165,8	37,4	6,1	8,0	0,860	0,646	0,945
	Knieextension (Nm)	256,0	62,2	261,4	61,7	5,0	8,8	0,933	0,829	0,973
	Knie-Flex/Ex-Quotient (%)	66,2	14,9	64,1	14,5	6,8	3,1	0,866	0,661	0,947

*ICC* Intraklassenkorrelationskoeffizienten, *M* Mittelwert, *SD* Standardabweichung, *SEM* Standard Error of the Measurement, *VK* Variationskoeffizient

**Abb. 3:**
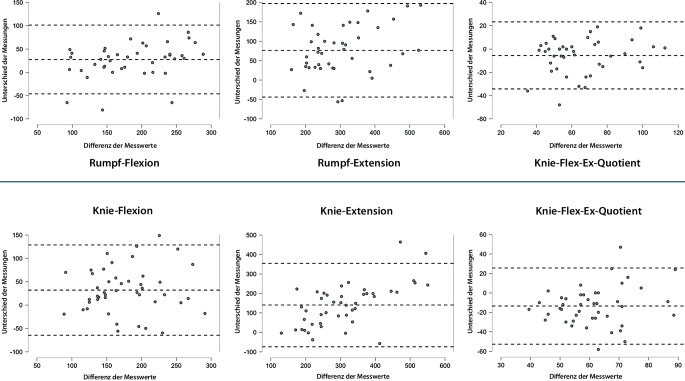
Bland-Altman-Plots (Bias ± LoA): Abweichungen der isometrischen (*Punktwolken*) in Relation zu den isokinetischen (*Null-Linienlevel*) Kraftkennwerten

Messwerte (Nm) der Rumpf- und Kniemaximalkraft waren bei den isometrischen Testungen grundsätzlich größer als in der konzentrisch-dynamischen (Quasi‑)Isokinetik (Tab. 3), und systemübergreifend waren die Kräfte (Momente, Nm) für die Extension größer als für die Flexion. Für das Verhältnis in der isometrischen Knietestung wurde eine Relation von etwa 1:2 (Flexion zu Extension: 54 %) ermittelt, während dieses Verhältnis für die isometrische Rumpfkrafttestung (62 %) und für die hydraulisch-isokinetischen Antagonisten-Quotienten für Rumpf und Knie (68 %) näherungsweise bei einem Verhältnis von 2:3 lag. Differenzen der Quotienten zwischen Isometrie und (Quasi‑)Isokinetik waren für den Rumpf weniger ausgeprägt als für den Kniequotienten (−5,5 % vs. −13,5 %) (Tab. 3). Unterschiede zwischen isometrisch und hydraulisch-isokinetisch ermittelten Kennwerten, aber auch die interindividuellen Variationen werden in Bland-Altman-Plots illustriert (Abb. 3). Die vorgestellten Orientierungswerte wurden auf die Körpermasse relativiert (Tab. 4).

**Tab. 3:** Bland-Altman-Statistiken (Bias, LoA) für Isometrie und Quasi-Isokinetik (*n* = 45)

	Isometrie		Isokinetik		Isometrie vs. Isokinetik		
	*M*	*SD*	*M*	*SD*	*Bias*	*Lower LoA*	*Upper LoA*
Rumpfflexion (Nm)	201,7	66,7	174,2	54,6	27,6	−46,2	101,3
Rumpfextension (Nm)	342,6	110,2	265,0	94,3	76,7	−43,8	197,2
Rumpf-Flex/Ex-Quot. (%)	62,5	21,5	68,0	19,0	−5,5	−34,2	23,3
Knie-Flex. (Nm)	192,3	55,8	160,3	53,1	32,0	−64,7	128,8
Knie-Ext. (Nm)	381,2	147,4	240,8	83,3	140,4	−73,7	354,4
Knie-Flex/Ex-Quot. (%)	54,5	16,5	68,0	13,7	−13,5	−52,6	25,6

*Bias* „mean difference“, *LoA* Limits of Agreement („mean difference“ −1,96*SD „lower LoA, and“ +1,96*SD „upper bound“)

**Tab. 4:** Orientierungswerte (pro kg KG) für Isometrie (oben) und Isokinetik (unten)

			M	SD	5 %	Q25 %	Med	Q75 %	95 %
Isometrie (Nm/kg KG)	Frauen	Rumpfextension	4,6	1,0	3,3	4,1	4,4	5,2	6,1
		Rumpfflexion	2,8	0,8	1,8	2,4	2,6	3,3	3,7
		Rumpf-Flex/Ex (%)	62,7	23,6	42,0	45,0	57,0	79,0	98,0
		Knieextension	4,8	1,6	2,7	3,7	4,6	6,0	7,5
		Knieflexion	2,5	0,5	2,0	2,2	2,4	2,7	3,1
		Knie-Flex/Ex (%)	57,2	19	31,0	42,0	56,0	65,0	82,0
		Beinextension (li)	30,2	6,5	16,0	19,0	23,1	31,3	44,7
		Beinextension (re)	23,1	5,6	15,5	18,5	22,9	26,0	32,1
		Symmetrie li/re (%)	8,5	8,3	0,9	1,0	6,0	13,5	17,8
	Männer	Rumpfextension	4,8	1,2	3,1	3,8	5,0	5,4	6,6
		Rumpfflexion	2,8	0,7	1,8	2,2	2,8	3,2	4,0
		Rumpf-Flex/Ex (%)	62,3	20,0	39,2	49,5	56,0	76,0	104,7
		Knieextension	5,6	1,5	3,6	4,3	5,2	6,8	8,0
		Knieflexion	2,8	0,6	1,8	2,4	2,8	3,2	3,6
		Knie-Flex/Ex (%)	52,2	14,1	35,5	44,0	48,5	56,0	80,4
		Beinextension (li)	22,2	6,5	14,5	16,3	20,7	28,8	31,4
		Beinextension (re)	23,8	6,4	14,6	17,1	24,0	30,0	33,2
		Symmetrie li/re (%)	11,3	9,5	0,0	5,0	9,0	17,5	28,5
Isokinetik (Nm/kg KG)	Frauen	Rumpfextension	3,4	0,8	2,2	3,0	3,7	3,9	4,6
		Rumpfflexion	2,3	0,5	1,4	2,0	2,2	2,5	3,1
		Rumpf-Flex/Ex (%)	70,1	22,3	43,0	53,0	64,0	85,0	108,8
		Knieextension	3,3	0,7	2,3	2,6	3,5	3,8	4,4
		Knieflexion	2,2	0,3	1,7	2,0	2,1	2,4	2,6
		Knie-Flex/Ex (%)	65,8	10,4	53,0	58,0	66,0	74,0	78,0
	Männer	Rumpfextension	3,8	1,0	2,7	3,2	3,7	4,6	5,2
		Rumpfflexion	2,9	0,5	1,7	2,1	2,7	2,8	3,1
		Rumpf-Flex/Ex (%)	66,0	15,9	46,1	53,5	63,0	78,5	89,5
		Knieextension	3,3	1,0	1,7	2,8	3,3	4,1	4,7
		Knieflexion	2,3	0,6	1,5	1,9	2,2	2,7	3,2
		Knie-Flex/Ex (%)	70,0	16,0	47,2	58,8	67,5	80,5	96,3

*Flex* Flexion, *Ex* Extension, *KG* Körpergewicht, *M* Mittelwert, *Med* Median, *SD* Standardabweichung, *Q* Quartile

Die Zeitnahme (Nebengütekriterium Zeitökonomie) während der Testsequenz ergab etwa 180 sec für das (quasi‑)isokinetische Protokoll (Flexion und Extension, inkl. Probezyklus und Pausen), während die nacheinander durchzuführende isometrische Testung (inkl. Probekontraktionen, mit Pausen) für Flexion und Extension auf etwa 360 sec kam.

## Diskussion

Ziel dieser Arbeit war es, die Vergleichbarkeit isometrischer und hydraulisch-gebremster isokinetischer Dynamometrie für die Flexion und Extension der Rumpf- und isolierten knieumspannenden Muskulatur zu evaluieren. Hierfür sollte als wissenschaftstheoretische Absicherung der notwendigen Testgüte zunächst die Test-Retest-Reliabilität überprüft und auch die Nebengütekriterien der Zeitökonomie sowie der Nützlichkeit (Orientierungsreferenzwerte) beachtet werden.

Die in der vorliegenden Arbeit ermittelten Reliabilitätskoeffizienten für die isometrische (ICC 0,61–0,90) und (quasi‑)isokinetische Testung (ICC 0,73–0,93) für Flexion und Extension lagen in etwa im Bereich dessen, was der gegenwärtige Stand der Literatur abbildet (ICC 0,87–0,96 isometrisch, respektive 0,69–0,99 isokinetisch) [6].

Es konnte lediglich eine Studie identifiziert werden, die eine spezielle hydraulisch-regulierte, konzentrisch-dynamische Krafttestung sowohl mit isometrischen als auch klassisch isokinetischen Tests (Biodex) gegenübergestellt – allerdings mit pneumatisch-hydraulischem Widerstand (Rehab exercise machine, HUR) [14]. Mit exzellenten Koeffizienten (ICC ≥ 0,98) war die Reliabilität hier noch höher als die Werte für die (quasi‑)isokinetische Knieextension und -flexion in der vorliegenden Arbeit (ICC 0,86–0,93) [14].

Die vorliegenden isometrischen Testungen der Rumpfflexion und Extension können direkt mit Factum®-Daten verglichen werden [7]. Die aktuell ermittelte Reliabilität war tendenziell niedriger (ICC Rumpf Extension 0,79 und Flexion 0,89) als jüngst publiziert (ICC Rumpf Extension 0,89 und Flexion 0,93) [7]. In dieser Arbeit aus dem Jahr 2024 wurde die knieumspannende Muskulatur einbeinig isometrisch ermittelt, während in der vorliegenden Arbeit beidbeinig getestet wurde. Die Reliabilität der beidbeinig getesteten isometrischen Knieflexion (ICC 0,87) war ähnlich hoch wie in der einbeinigen Testung (ICC 0,88–0,98), während dies für die beidbeinig-isometrische Extension (ICC 0,61) bedeutend niedriger war als vormals einbeinig ermittelt (ICC 0,93–0,95) [7].

Ähnliches gilt für die Reliabilität der funktionellen Antagonistenquotienten der isometrisch ermittelten Rumpf- und Kniekraft, wo vorliegende Werte (ICC Rumpfquotient 0,89, bzw. Kniequotient 0,55) sich vor allem im Kniequotienten von vormals ermittelten Daten (ICC Rumpf 0,87, bzw. Knie 0,38–0,92) unterschieden [7]. Hydraulisch-isokinetisch wurde für den Knieantagonistenquotienten jedoch eine höhere Reliabilität ermittelt (ICC 0,87) als für klassisch isokinetische Dynamometer (Cybex Model 770, Biodex System 3 Pro: ICC 0,62–0,73) [15].

Die einbeinige Hüft-Knie-Streckung (Funktionsstemme) wurde sehr verlässlich ermittelt (ICC 0,92–0,95), während der Symmetriequotient mit Links-Rechts-Differenzen von 11,2 % (Test), bzw. 7,5 % (Retest) als schwächer reproduzierbar einzustufen ist (ICC 0,65). Für die Praxis ist diese schwächere Reliabilität nicht unproblematisch, weil Seitendifferenzen von 10–15 % als therapie- bzw. ausgleichsbedürftig eingestuft werden [16], was durch unzuverlässig ermittelte Quotienten korrumpiert werden kann.

Während für den Seitenvergleich ein idealisiertes Verhältnis von 1:1 (links-rechts) mit akzeptablen Abweichungen von etwa 10 % angenommen werden darf [16], ist dies bei Quotienten zur Beschreibung eines ausbalancierten Normalzustandes der funktionellen Antagonisten (Flexion-Extension) nicht so eindeutig. Ausgleichsmaßnahmen sind vorzuschlagen, wenn für das jeweilige Messsystem eigene verlässliche Orientierungswerte vorliegen. Gerätespezifisch können jedoch unterschiedliche isometrische Verhältnisse als ausbalanciert beobachtet werden (Rumpf 58–37 %, Knie 41–57 %) [7].

In der vorliegenden Arbeit wird für beschwerdefreie Jungerwachsene ein ausbalanciertes Flexion-zu-Extension-Verhältnis von 63 %, bzw. 68 % für die isometrisch bzw. (quasi‑)isokinetisch getesteten Rumpfantagonisten angegeben, und 55 %, bzw. 68 % für die entsprechend getesteten kniegelenkumspannenden Antagonisten.

Der Flexion-Extension-Quotient in der (quasi‑)isokinetischen Rumpftestung ist somit in etwa übertragbar auf den isometrischen Rumpfquotienten – beide weisen ein vergleichbar ausbalanciertes Verhältnis von annähernd 2:3 auf (Isometrie 5,5 % niedriger als Isokinetik; Tab. 3). Für den Kniequotienten gilt das nicht – das ausbalancierte Verhältnis ist hier (quasi‑)isokinetisch zwar auch etwa 2:3, aber isometrisch ist diese Relation in etwa 1:2 (Isometrie 13,5 % niedriger als Isokinetik; Tab. 3), wobei zusätzlich geschlechtsspezifische Unterschiede zu beachten wären (Tab. 4).

Die aktuellen Befunde bestätigen frühere Arbeiten, wonach Orientierungswerte für jedes Messsystem zu erheben sind, weil eine systemübergreifende Vergleichbarkeit weder für die Isometrie noch für die Isokinetik angenommen werden darf [7, 13]. Trivial ist hierbei, dass isometrisch ermittelte Drehmomente höher sind als korrespondierende isokinetische Werte – zusätzlich notwendige Kräfte zum Überwinden äußerer Widerstände in der Isokinetik fließen in der Isometrie als höhere Maximalkraftwerten ein [16].

Die vorliegenden Orientierungsdaten – insbesondere die nonparametrischen Kennwerte (Perzentile) der auf das Körpergewicht relativierten Orientierungswerte – stehen zur klinischen Einordnung von Patientenkraftkennwerten zur Verfügung ([7]; Tab. 4). Für wissenschaftlich-akademische Nutzungen können auch unadjustierte Werte interessieren (*Supplement 2*).

Durch die parallel erhobenen hydraulisch-isokinetischen Kennwerte für Flexion und Extension ist die Zeitökonomie in Relation zur Isometrie etwa doppelt so effizient; anders im Vergleich zur klassischen, elektromechanisch gesteuerten Isokinetik [6]. Die ausschließlich konzentrische Testung in der (Quasi‑)Isokinetik erscheint für Rehabilitationsprozesse in der Allgemeinbevölkerung angemessen. Aber dieser Zeitökonomievorteil steht einem inhaltlichen Nachteil gegenüber, wenn zum Beispiel im leistungssportlichen Umfeld zur Abschätzung von Knieverletzungsrisiken auch die exzentrisch realisierbaren Kräfte der Hamstrings interessieren, um Quotienten aus exzentrischer Beuge- und konzentrischer Streckkraft zu nutzen [17].

## Ausblick

Orientierungsdatenbanken sollten in zukünftigen Studien um weitere Muskelgruppen ergänzt werden – insbesondere für die hydraulische Isokinetik, für die trotz vielversprechender Testökonomie bislang kaum Daten vorliegen.

## Fazit für die Praxis

- Die Reliabilität (Hauptgütekriterium) ist für beide Messsysteme akzeptabel – trotz der Unterschiede zwischen Isometrie und Isokinetik.
- Insbesondere bei der Kniefunktion sind die errechneten funktionellen (Flex/Ex)-Quotienten nicht miteinander vergleichbar.
- Orientierungswerte für den klinischen Einsatz stehen zur Verfügung.
- Vorteil (Quasi‑)Isokinetik: Zeitökonomie (Nebengütekriterium).
- Vorteil (Quasi‑)Isokinetik: Transfer Test-Training – Ableitung der Trainingsintensität aus der Testung und verlässliche Abbildung von Leistungsänderungen im Kraftmonitoring.

## Supplementary Information

Illustrationen und präzise Beschreibungen zur Positionierung der Testpersonen für die Standardisierung der Gelenkausgangsstellungen und zur Übereinstimmung von Geräte- und Gelenkdrehachsen für eine zuverlässige Krafttestung

Tabellarische Aufbereitung der absoluten, d. h. nicht auf das Körpergewicht relativierten Drehmomente

## Data Availability

Die in dieser Studie erhobenen Datensätze können auf begründete Anfrage beim Korrespondenzautor angefordert werden.

## Abkürzungen

BMI: Body Mass Index
CI: Confidence Interval
DMS: Dehnungsmessstreifen
Hz: Hertz
ICC: Intra Class Correlation
KH: Körperhöhe
KM: Körpermasse
LoA: Limits of Agreement
M: Mittelwert
min: Minute
Nm: Newtonmeter
Q: Quartil (25%, bzw. 75% Quartil)
SD: Standardabweichung
SEM: Standard Error of the Measurement
t1: Zeitpunkt 1
t2: Zeitpunkt 2

## Footnotes

1 Im Gegensatz zu elektronisch gebremsten isokinetischen Testsystemen waren bei der vorliegenden hydraulischen Widerstandsgebung keine exzentrisch-nachgebenden Test- und Trainingsbewegungen möglich, sodass hier von einer „quasi-isokinetischen“ Testapparatur gesprochen werden soll.
