# Supplementary figures and images for: Hydraulically controlled isokinetic strength testing
Source: Orthopadie (Heidelb). 2025 Jul 17;54(10):785–94. [Article in German] doi: 10.1007/s00132-025-04670-3 (PMC12457523; doi:10.1007/s00132-025-04670-3)

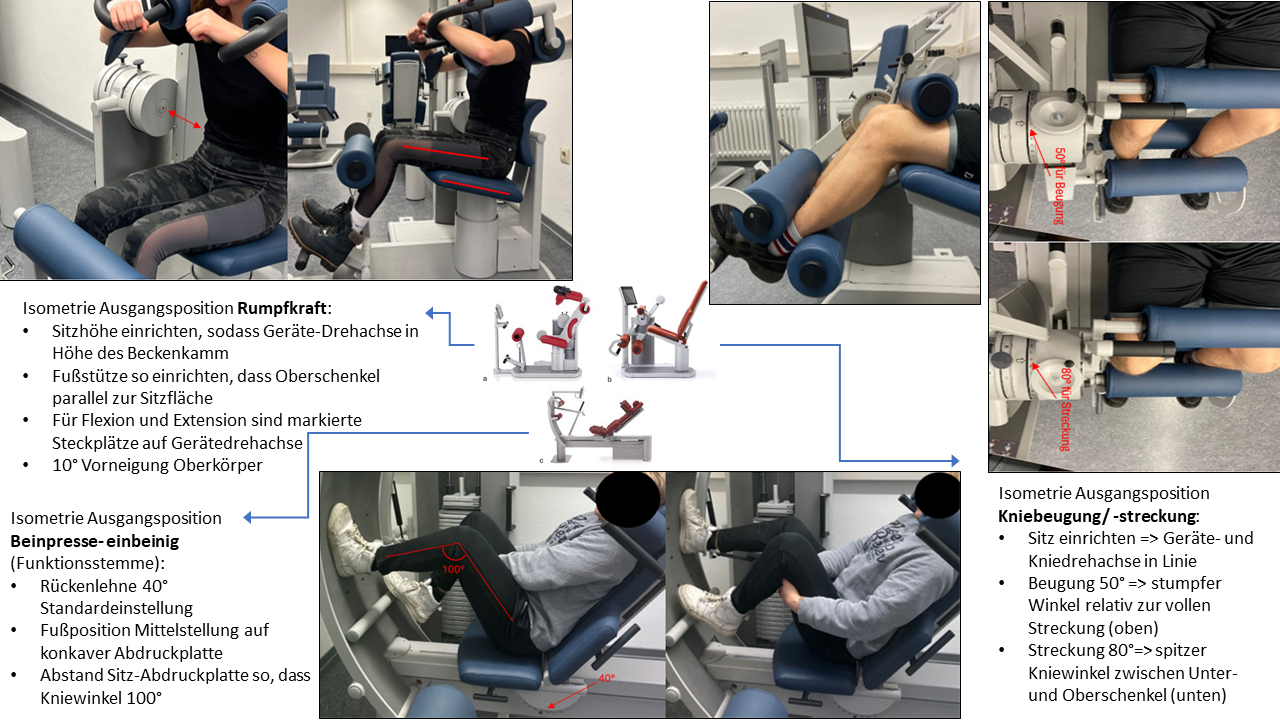

Supplement: Supplementary file 1 — Illustrationen und präzise Beschreibungen zur Positionierung der Testpersonen für die Standardisierung der Gelenkausgangsstellungen und zur Übereinstimmung von Geräte- und Gelenkdrehachsen für eine zuverlässige Krafttestung [file 132_2025_4670_MOESM1_ESM.tif]
