# Supplementary material for: Hydraulically controlled isokinetic strength testing
Source: Orthopadie (Heidelb). 2025 Jul 17;54(10):785–94. [Article in German] doi: 10.1007/s00132-025-04670-3 (PMC12457523; doi:10.1007/s00132-025-04670-3)
Supplement: Supplementary file 2 — Tabellarische Aufbereitung der absoluten, d. h. nicht auf das Körpergewicht relativierten Drehmomente [file 132_2025_4670_MOESM2_ESM.pdf]

Tab. S2 – Orientierungswerte (absolut) für Isometrie (oben) und Isokinetik (unten)

			M	SD	5%	Q25 %	Med	Q75%	95%
Isometrie (Nm)	Frauen	Rumpfextension	286,4	62,6	183,0	258,0	281,0	330,0	362,0
		Rumpfflexion	173,1	57,0	99,0	151,0	169,0	196,0	270,0
		Rumpf-Flex/Ex (%)	62,7	23,6	42,0	45,0	57,0	79,0	98,0
		Knieextension	296,0	99,1	177,0	206,0	265,0	377,0	464,0
		Knieflexion	154,4	32,5	111,0	143,0	155,0	178,0	207,0
		Knie-Flex/Ex (%)	57,2	18,9	31,0	42,0	56,0	65,0	82,0
		Beinextension (li)	30,2	27,6	16,0	19,0	23,0	31,2	44,7
		Beinextension (re)	23,0	5,6	15,5	18,4	22,8	26,0	32,0
		Symmetrie li/re (%)	8,5	8,3	0,9	1,0	6,0	16,5	17,8
	Männer	Rumpfextension	394,0	120,0	234,1	283,5	394,0	472,0	586,5
		Rumpfflexion	227,8	65,2	114,1	193,5	233,0	276,0	308,9
		Rumpf-Flex/Ex (%)	62,3	20,1	39,2	49,5	56,0	76,0	104,7
		Knieextension	455,7	143,5	286,6	349,0	420,0	554,5	699,2
		Knieflexion	255,5	50,8	142,1	198,5	222,0	256,8	310,2
		Knie-Flex/Ex (%)	52,2	14,2	35,5	44,0	48,5	56,0	80,4
		Beinextension (li)	22,1	6,5	14,5	16,3	20,7	28,7	31,3
		Beinextension (re)	23,8	6,4	14,6	17,1	24,0	29,9	33,2
		Symmetrie li/re(%)	11,3	9,5	0,0	5,0	9,0	17,5	28,5
Isokinetik (Nm)	Frauen	Rumpfextension	211,7	51,7	146,0	181,0	210,0	259,0	277,0
		Rumpfflexion	141,1	35,7	78,9	125,0	140,0	161,0	204,0
		Rumpf-Flex/Ex (%)	70,0	22,2	43,0	53,0	64,0	85,0	108,0
		Knieextension	206,0	46,1	132,4	166,0	208,0	230,0	277,0
		Knieflexion	133,4	23,4	98,5	121,0	130,0	140,0	183,0
		Knie-Flex/Ex (%)	65,8	10,4	53,0	58,0	66,0	74,0	78,0
	Männer	Rumpfextension	315,4	97,9	175,1	249,5	321,0	389,0	444,9
		Rumpfflexion	204,4	51,6	112,3	175,5	217,0	237,5	269,0
		Rumpf-Flex/Ex (%)	66,0	15,8	46,1	53,0	63,0	78,5	89,5
		Knieextension	271,2	96,7	131,5	198,0	271,0	340,5	422,7
		Knieflexion	183,8	60,6	90,8	144,8	189,0	224,0	260,0
		Knie-Flex/Ex (%)	70,0	16,0	47,2	58,8	67,5	80,5	96,3

Legende: *Flex* Flexion, *Ex* Extension
